# Measurement of wind field data in Southeast China

**DOI:** 10.1016/j.dib.2018.09.082

**Published:** 2018-10-02

**Authors:** Li Lin, Kai Chen, Dandan Xia, Huaifeng Wang, Haitao Hu, Fuqiang He

**Affiliations:** a Xiamen University of Technology, China; b Fujian Jiadesign Co. LTD. China; c University of Regina, Canada

## Abstract

The data presented in this article are the wind measurements acquired from a tower in Southeast China during typhoon Nesat (1709#) and typhoon Haitang (1710#). Three 3D ultrasonic anemometers Wind Master Pro were utilized to obtain 3D wind data. The anemometer works well with wind speed range of 0–65 m/s and wind angle range of 0–360°. Three direction wind speeds and wind angles were recorded per every 0.1 s. The present research analyzed wind characteristics based on recorded data. In this article, the detailed test set-up and data pre-processing methodology for the wind characteristics analysis are provided.

**Specifications table**

**Table:** 

Subject area	*Civil Engineering*
More specific subject area	*Wind engineering, aerodynamics,*
Type of data	*Table, graph, figure*
How data was acquired	3D ultrasonic anemometer Wind Master Pro, collection system CR3000
Data format	*Filtered, analyzed*
Experimental factors	*3-direction wind speeds are recorded during two typhoons. Both wind speed and wind angle are recorded*
Experimental features	*Full scale measurements are performed; sensors are installed to record wind speed and wind angle; both seasonal and typhoon wind speed are obtained*
Data source location	*Pingtan County, Fujian Province, China* (119°52′23″E, 25°33′24″N)
Data accessibility	*Data is with this article*

**Value of the data**

• The data can be used for the analysis of wind characteristics under typhoon climate in southeast China.
• The full-scale measurement data can be used by wind tunnel experimenters for validating or justifying their testing results.
• The wind speed data can be used by CFD model developers for validating their numerical results or determining the boundary conditions to be used.
• The wind characteristics analysis based on the recorded wind speed data may provide references for designing the wind-resistant structures to be used in southeast China.

## 1 Data

**Fig. 1:**
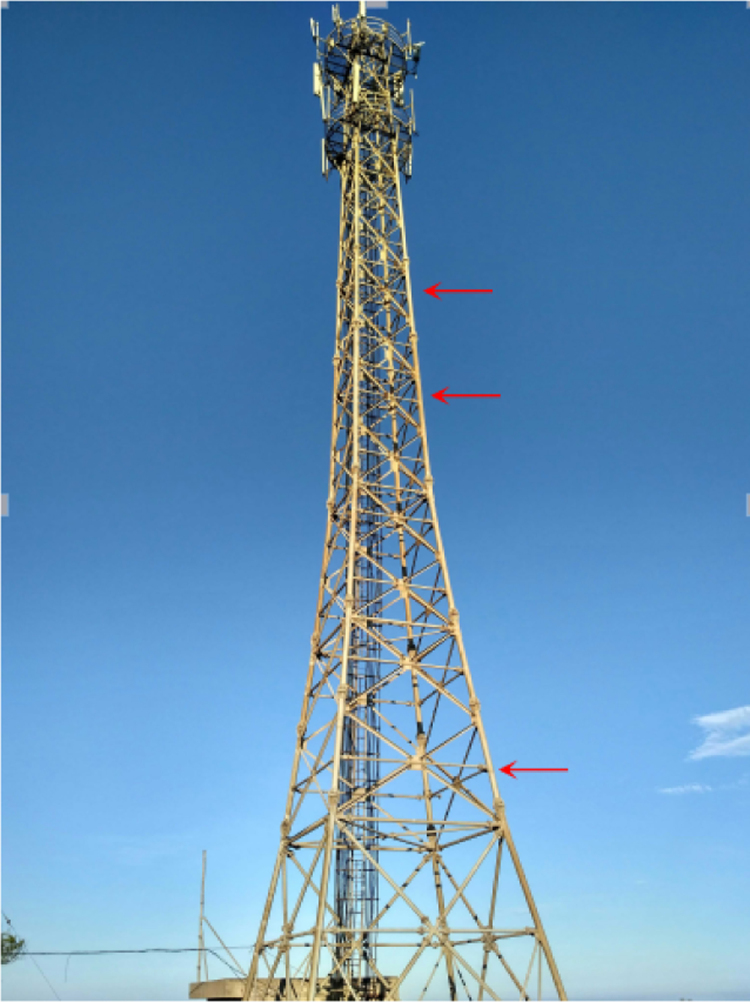
Schematic of experiment set up.

The data presented in this article is acquired from the wind field measurement station (as shown in Fig. 1) at Pingtan in Southeast coastal of China when Typhoon Nesat (1709#) and Typhoon Haitang (1710#) attacked Pingtan during 30th and 31st of July in 2017. The recorded wind speed data during typhoon process is obtained. To ensure the data quality, the wind speed data were filtered by data control method. The filtered wind speed data during typhoon is available in the supplementary material. The filtered data was analyzed to obtain wind characteristics presented in paper “Analysis on the Wind Characteristics under Typhoon Climate at the Southeast coast of China.”

## 2 Experimental design

### 2.1 Experiment set up

**Fig. 2:**
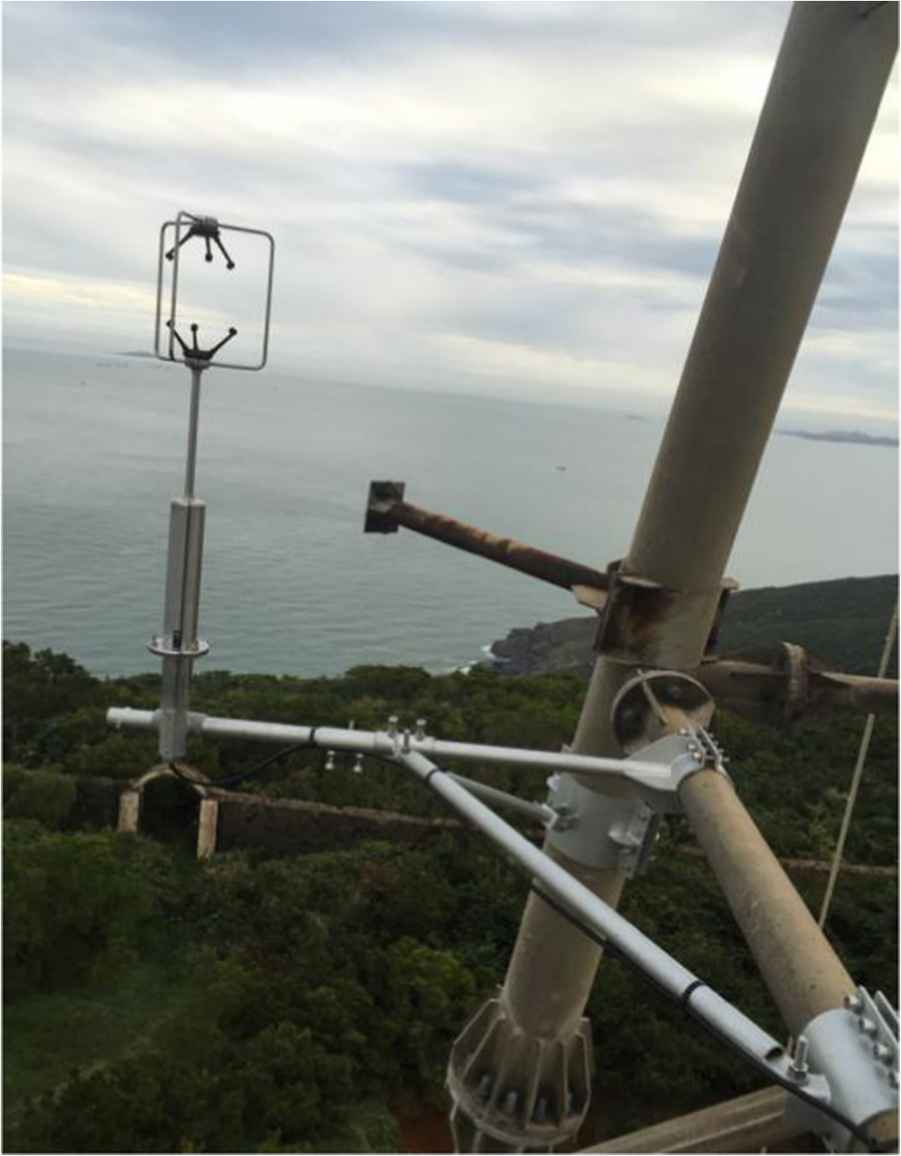
Photo of installation.

**Fig. 3:**
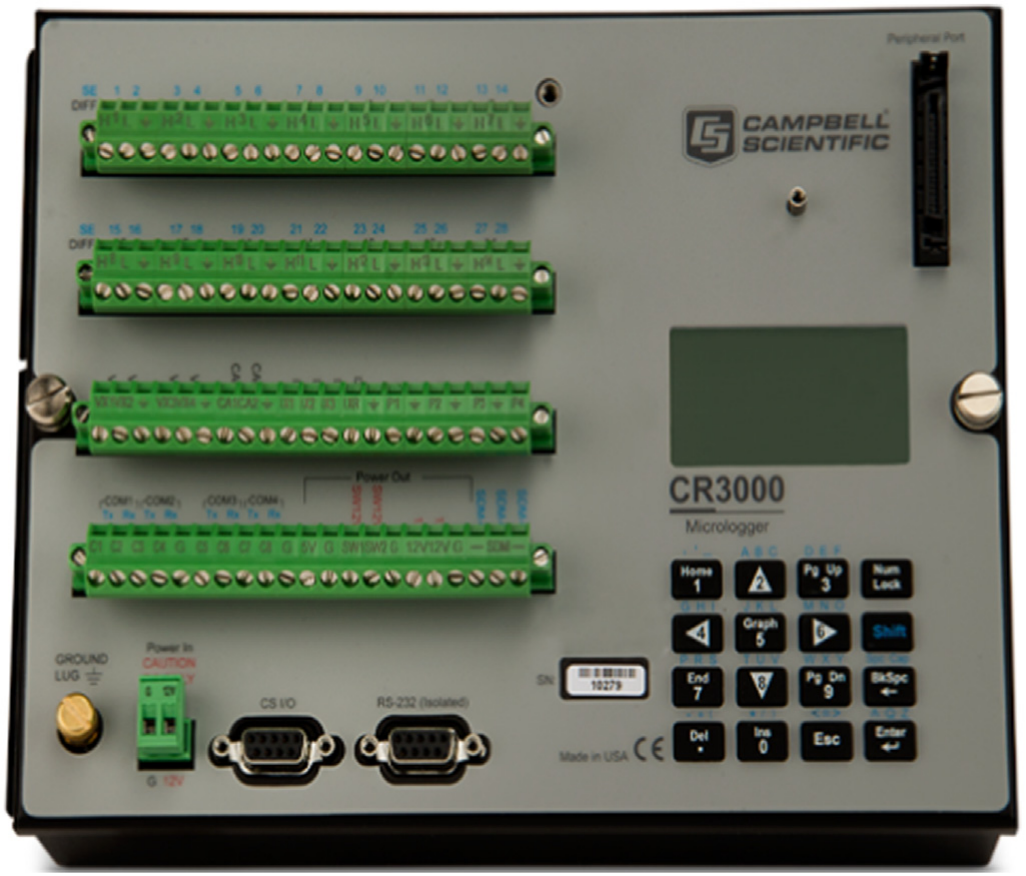
Photo of collection system.

The experiment station was set up in Pingtan, Fujian Province, China. The location of sensors installation can be seen in the Fig. 1. There 3D ultrasonic anemometers Wind Master Pro produced by Gill Company in UK, were installed for the record of wind speed data. The arrangement of sensors can be seen in the Table 1. The corresponding main parameters are: wind speed range: 0–65 m/s; resolution ratio: 0.01 m/s; wind direction range: 0–359°; resolution: 0.1° and frequency 10 Hz. The anemometers were installed on the tower by the designed steel holder as shown in Fig. 2. The data was collected and monitored by data acquisition system CR3000 as can be seen in the Fig. 3.

**Table 1:** Arrangement of anemometers.

Anemometer type	Installation height (m)
141,703	10
151,906	26
160,210	32

### 2.2 Method

**Fig. 4:**
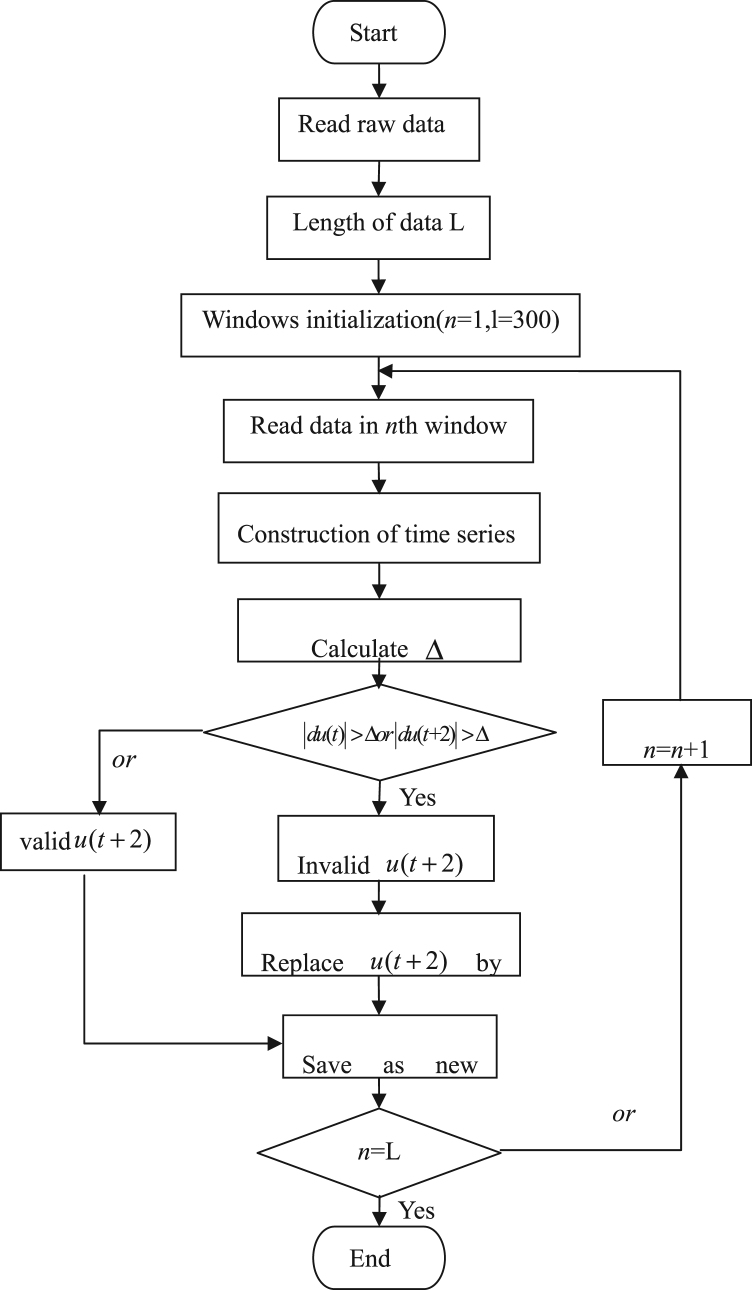
Flow chart of data control method.

Based on the above observation station, the wind speed data were obtained during typhoon Nesat and typhoon Haitang. However, the recorded wind speed data may include some bad or invalid data. The record data was filtered to ensure the validation by data controlled method (Fig. 4). The reliability of data was firstly diagnosed by comparing experienced wind speed observed by nearby meteorological station. A multiple truncation variance method [1], [2], [3] was used to determine the rationality of the original data. The smooth estimation of the original data was performed for each time series (30 s). By detecting the sudden change of the data, it was determined whether the value exceeded the range of the smooth estimation to ensure the validity of the data point. Data processing can be specified as follows:

Calculate the time series du(t) as:

(1) du(t)=u(t+2)−u(t)

The mean value of du(t) and $du^{2}$ are:

(2) $$\overset{¯}{du}=\frac{1}{n-2}\sum_{i=1}^{n-2}du(t),\;\overset{¯}{du^{2}}=\frac{1}{n-2}\sum_{i=1}^{n-2}du{\left(t\right)}^{2}$$

The truncation variance can be expressed as follow:

(3) $$\sigma =\overset{¯}{du^{2}}-{\overset{¯}{du}}^{2}$$

The criterion to detect invalid data can be defined as:

(4) $$\Delta =c\cdot \sigma^{0.5}$$

In this research, u(t) is the wind speed at *t*th time point, in the Eq. (4), *c* = 4, which means when the absolute value of the difference between the mean value of the sample point and the total sample is greater than 4 times the standard deviation, the point will be diagnosed as unreasonable data and need to be modified. The modification process can be seen as in Fig. 4, Five-point interpolation method was applied as indicated in Eq. (5). The procedure of data quality control can be seen in the Fig. 4.

(5) $$u^{\left(3\right)}=\frac{1}{4}(u_{t+1}^{\left(2\right)}+2u_{t+2}^{\left(2\right)}+u_{t+3}^{\left(2\right)})$$

where $u^{\left(1\right)}$ is the median of the five data points u(t+i)(i=0...4), $u^{\left(2\right)}$ is the median of $u_{t+1}^{\left(1\right)}$ and $u_{t+3}^{\left(1\right)}$.

Moreover, to avoid the noise during collection process, the data was low-pass filtered at 3 Hz. Filtered data are available in the supplementary material which can be used for the wind characteristics analysis.
