# Supplementary material for: Measurement of wind field data in Southeast China
Source: Data Brief. 2018 Oct 2;21:328–33. doi: 10.1016/j.dib.2018.09.082 (PMC6197946; doi:10.1016/j.dib.2018.09.082)
Supplement: Supplementary file 1 — Supplementary material. [file mmc1.doc]

**Disclosure statement**

No potential conflict of interest was reported by the authors
